# Supplementary material for: Ancestral Hybridization Facilitated Species Diversification in the Lake Malawi Cichlid Fish Adaptive Radiation
Source: Mol Biol Evol. 2019 Dec 14;37(4):1100–13. doi: 10.1093/molbev/msz294 (PMC7086168; doi:10.1093/molbev/msz294)
Supplement: msz294-Supplementary_Data [file msz294-supplementary_data.zip › msz294-Suppl_Data/Svardaletal_MBE_201911_supplementary_text_and_figures.pdf]

Supplementary materials for

Ancestral hybridisation facilitated species diversification in the Lake Malawi cichlid fish adaptive radiation

Hannes Svardal, Fu Xiang Quah, Milan Malinsky, Benjamin P Ngatunga, Eric A Miska, Walter Salzburger, Martin J Genner, George F Turner, Richard Durbin

Simulations

We performed neutral coalescent simulations using msprime (Kelleher et al. 2016) to test whether the observed excess-divergence of hybridisation-derived variation is observed under neutral evolution. The simulations were designed to qualitatively match the diversity patterns observed in the real data (supplementary fig. S4).

In particular, within-sample effective population sizes were inferred from real data as $N_e = \pi/(4\mu)$, where π is the number of pairwise genetic differences per accessible site in the genome, and μ is the per generation mutation rate set to 3*3.5e-9 as inferred in (Malinsky et al. 2018) and assuming a generation time of three years. Since alignment here was performed against the relatively distant outgroup genome Orenil. 1.1 (Tilapia) with an average pairwise sequence divergence of ~6% over fourfold degenerate sites in Brawand et al. (2014), and conservative genome filtering was performed (see Materials and Methods), there is a certain bias in our accessible genome to overall more conserved genomic regions. Consistent with this, divergence to the reference genome in our accessible genome is only ~3%. To account for this, we scaled all branch length estimates with a scaling factor of 1.53, which scales the date of the split between pelagic (*Rhamphochromis*, *Diplotaxodon*) and other Lake Malawi cichlid species to the 460 thousand years estimated by Malinsky et al. (2018).

Pairwise split times of two lineages *x* and *y* were estimated as

$$T_{split} = \frac{d_{xy} - (\pi_x + \pi_y)/2}{2\mu}, \quad (1)$$

where d_{xy} is cross-lineage pairwise sequence divergence per accessible site. A split tree was constructed from these data using the neighbour-joining algorithm. The unequal length of terminal branches, which could be the result of variation in mutation rates, generation time, or

drift was implemented in the simulations by setting time zero for the longest branch and treating shorter branches as ancient samples (times given in fig. S4).

Effective population sizes on internal branches were set to the average of the two child branches. Note that for computational efficiency, in the simulations the non-Malawi lineages *Victoria*, *Ruaha Blue*, *A. gigliolii*, and *H. vanheusdeni* were each represented by panmictic (meta)populations with effective population sizes set to match the overall diversity patterns in these (inhomogeneous) groups. For these branches, when calculating N_e of parental branches, the average of the actual sample effective population sizes was used rather than the N_e of the metapopulation.

Two instantaneous gene flow events were added to the simulations: Gene flow into the common ancestor of the Malawi radiation from the *Ruaha Blue* branch as inferred in fig. 2, and, within *Malawi*, gene flow from the pelagic clade *Diplotaxodon* into the benthic clade, which constitutes the strongest gene flow event among major *Malawi* groups inferred in Malinksy et al. (2018). Note that simulation input gene flow fractions of 22% and 31%, respectively, are set to match inferred f_4 admixture fractions of 10% each. Furthermore, we lengthened the branch of the ancestor of the non-pelagic Malawi clade by 10k generations, because an expected effect of the observed gene flow is to shorten this branch, and otherwise benthic species cluster with the pelagics in the simulation results.

The simulation results give a reasonable match to the data (supplementary figs. S2, S6, S7). Cross-species pairwise differences and F_{ST} are slightly higher in the simulations than in the real data. This can be explained by additional gene flow events between Malawi species that have not been modelled here (see Malinsky et al. 2018) leading to larger amounts of shared variation between non-sister-branches.

In the neutral simulations, the proportion of F_{ST} outliers is elevated for both old shared variation and hybridisation-derived variation relative to private *Malawi* variants for low heterozygosity bins (fig. S12, right panel). But importantly, there is no excess divergence of hybridisation-derived variants as compared to old shared variation. For the heterozygosity bins 0.2-0.3 and higher, there is no significant difference between any of the variant categories. The difference between private and non-private variation in the heterozygosity bins 0-0.1 and 0.1-0.2 is due to the fact that non-private variants are relatively enriched at the right end of these bins.

Supplementary Figures

FIG. S1

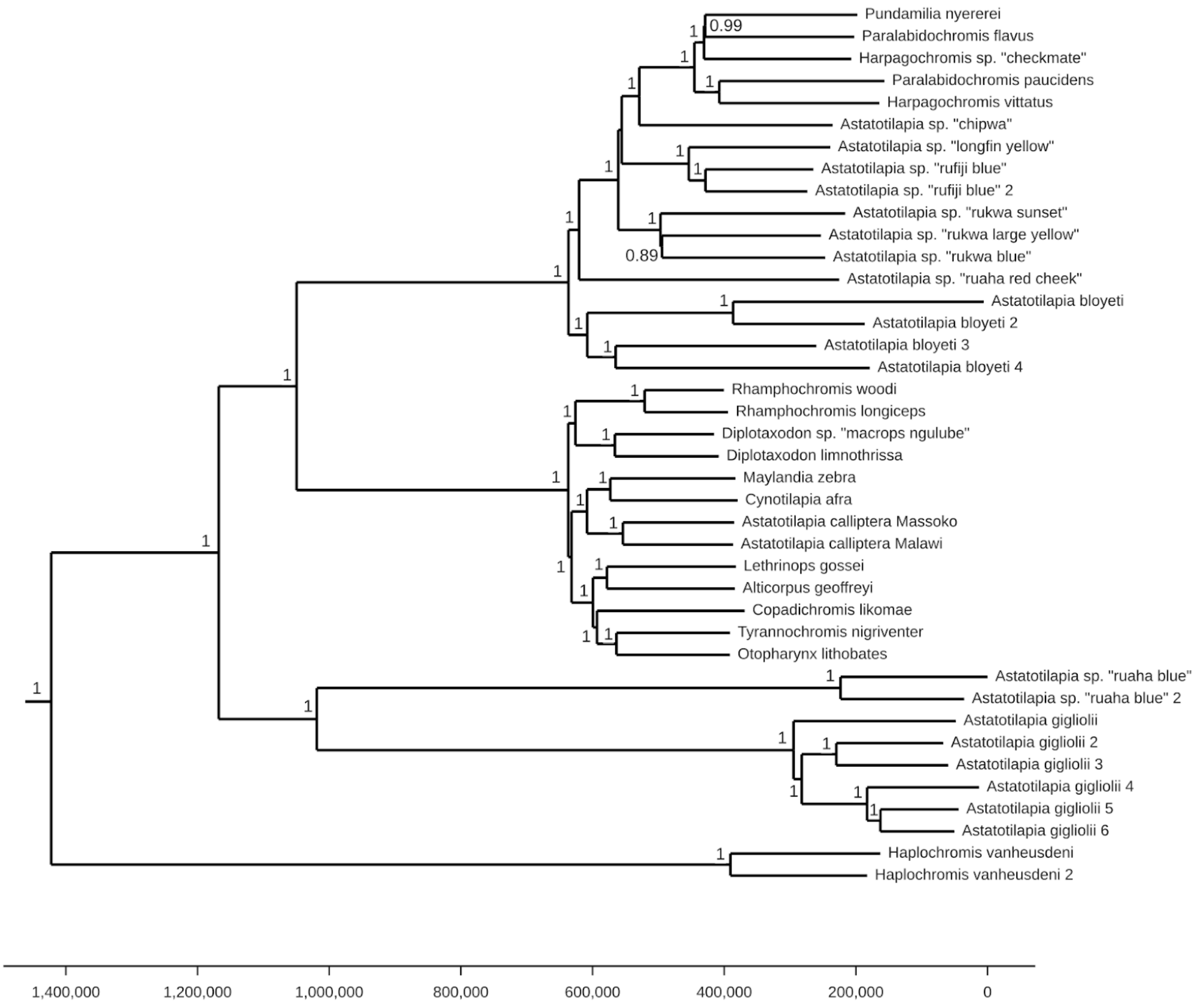

Neighbour joining sample relatedness tree with block-bootstrap support values (black numbers). Bootstrap support was inferred from 100 bootstrap re-samples of pairwise differences calculated in 1000 SNP windows (Materials and Methods). The scale bar gives the absolute count of pairwise differences.

FIG. S2

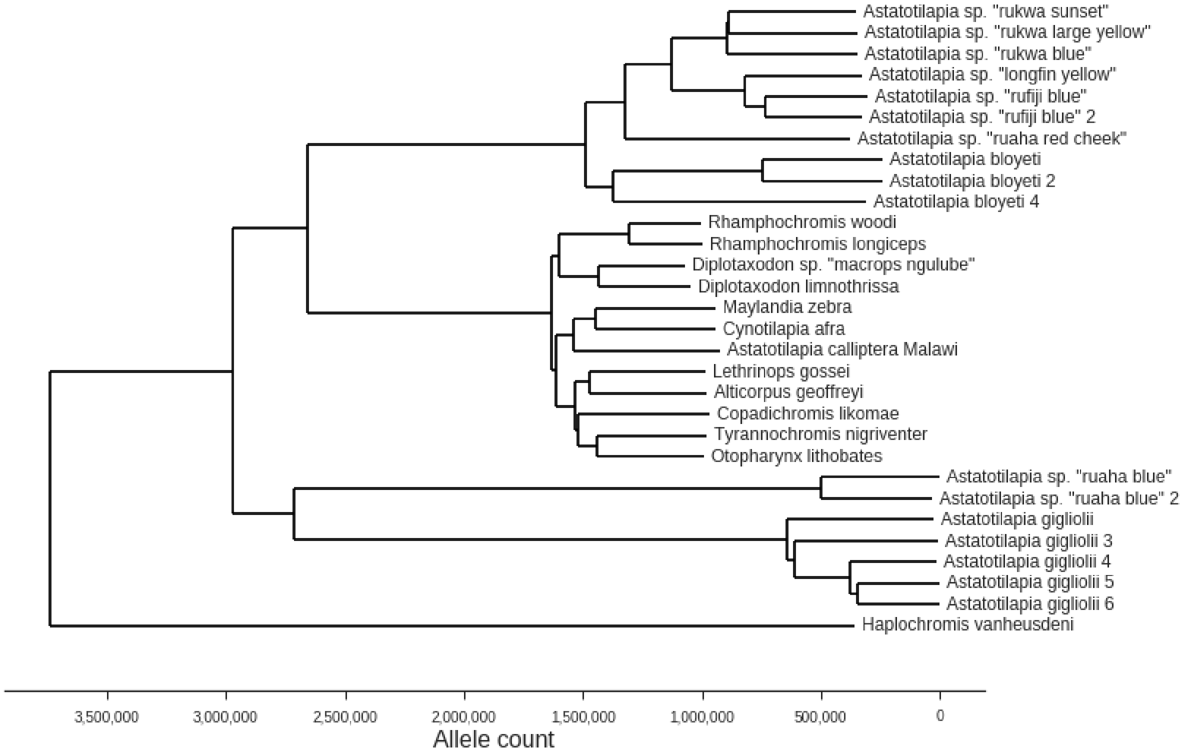

Neighbour joining sample relatedness tree for a subset of the samples that was realigned to a reference genome of *Astatotilapia calliptera* (GCA_900246225.3, https://www.ncbi.nlm.nih.gov/assembly/GCF_900246225.1/) (Materials and Methods). All topological relationships are consistent with the tree based on the alignment to the outgroup *Oreochromis niloticus*. The larger absolute numbers of differences compared to fig. S1 are due to a larger accessible genome size of 653 megabases.

FIG. S3

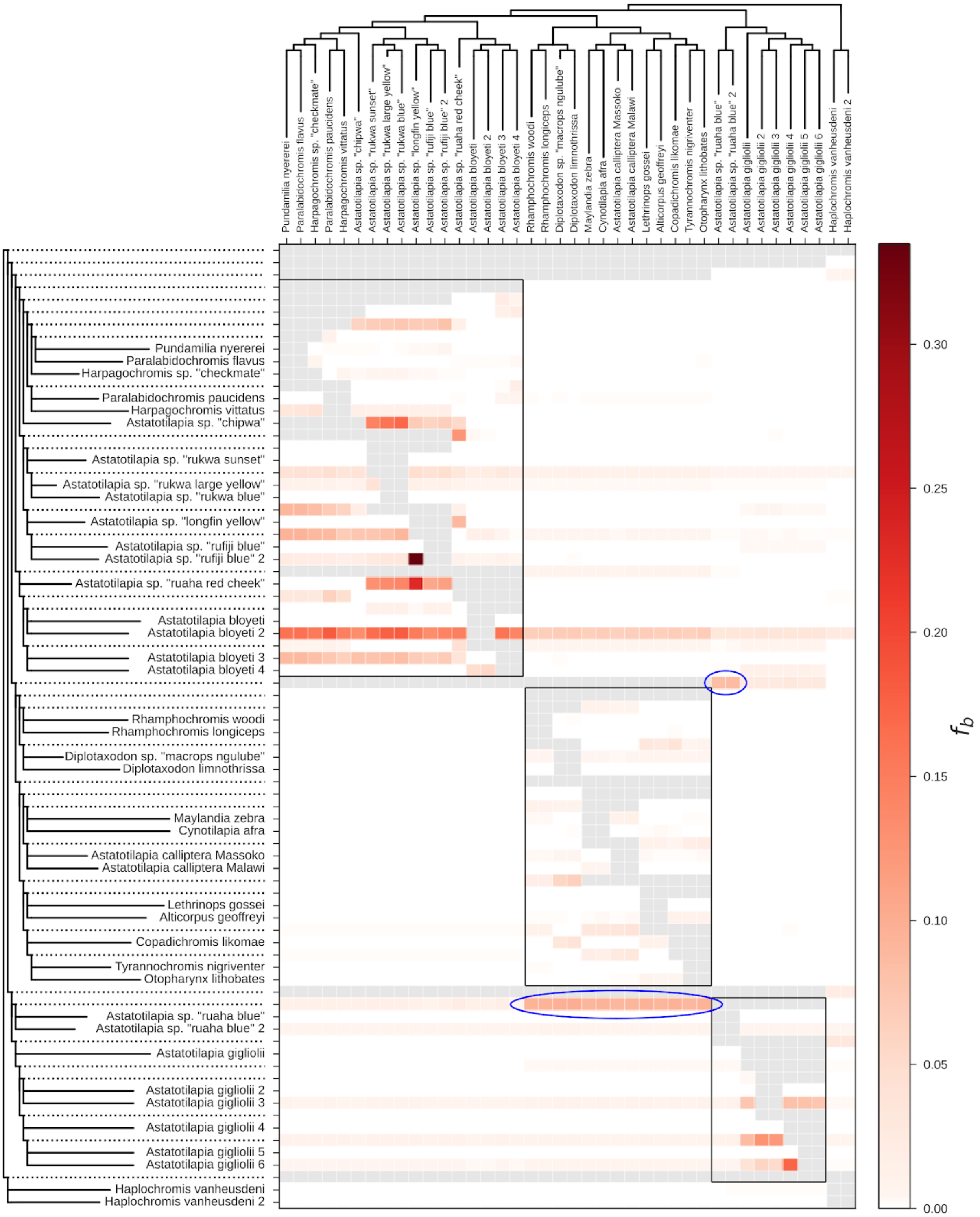

Excess allele sharing between samples. The f_b statistic is a summary of f4 admixture ratios and shows excess allele sharing between the branch on the y-axis and the sample on the x-axis (Malinsky et al. 2018). Grey colour correspond to tests that are not possible. Large black rectangles mark comparisons within the named clades in fig. 1b. The strongest signal of cross-clade excess allele sharing is between the common ancestor of the Ruaha Blue samples and the Malawi samples (blue circles). *A. bloyeti* 2 shows excess alle sharing with all other samples compared to its sister branch *A. bloyeti*. This is consistent with gene flow

from an unsampled taxon that constitutes an outgroup to all sampled taxa into *A. bloyeti*. The complete set of f4 admixture ratios that are consistent with the nj-tree are given in supplementary table 2.

FIG. S4

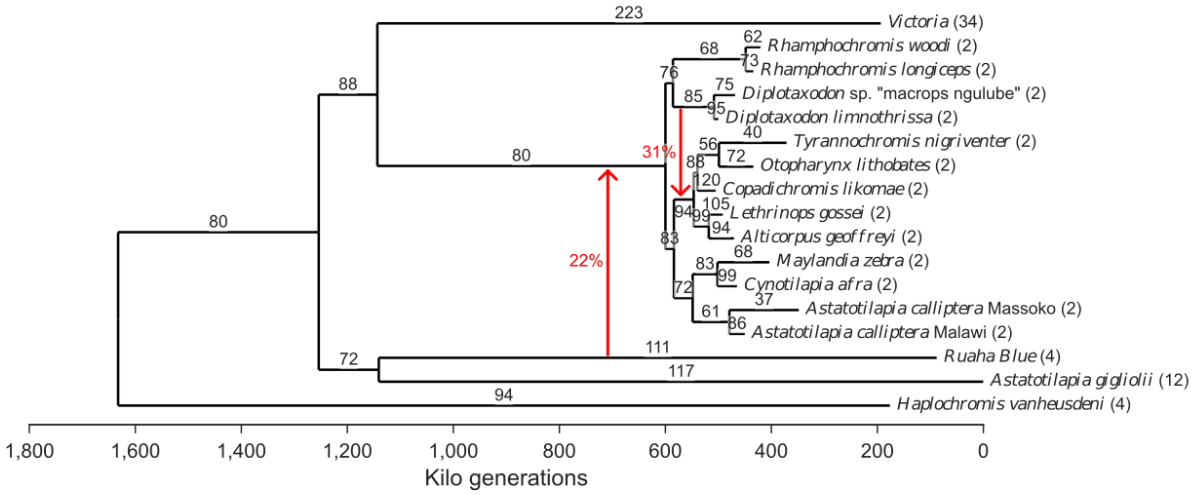

Input species split tree for the coalescent simulations in msprime (Kelleher et al. 2016). Numbers on branches indicate piecewise-constant effective population sizes in thousands. Red arrows and associated percentages indicate gene-flow events ("MassMigrations" in msprime). Numbers in parenthesis right of sample names indicate haploid sample sizes.

FIG. S5

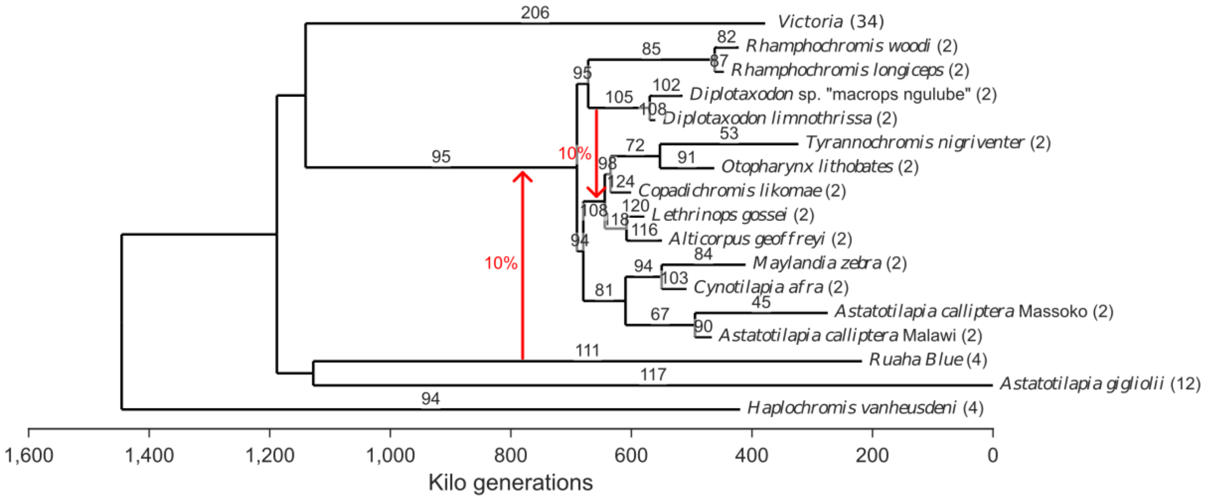

Species tree inferred from the coalescent simulations (see fig. S4 for simulation input). Numbers on branches indicate piecewise-constant effective population sizes. Red arrows and associated percentages indicate gene-flow proportions inferred using the f_4 admixture ratio. Numbers in

parenthesis right of sample names indicate haploid sample sizes. Note that the split time of the outgroup *H. vanheusdeni* cannot be inferred from the data and is set posteriori.

FIG. S6

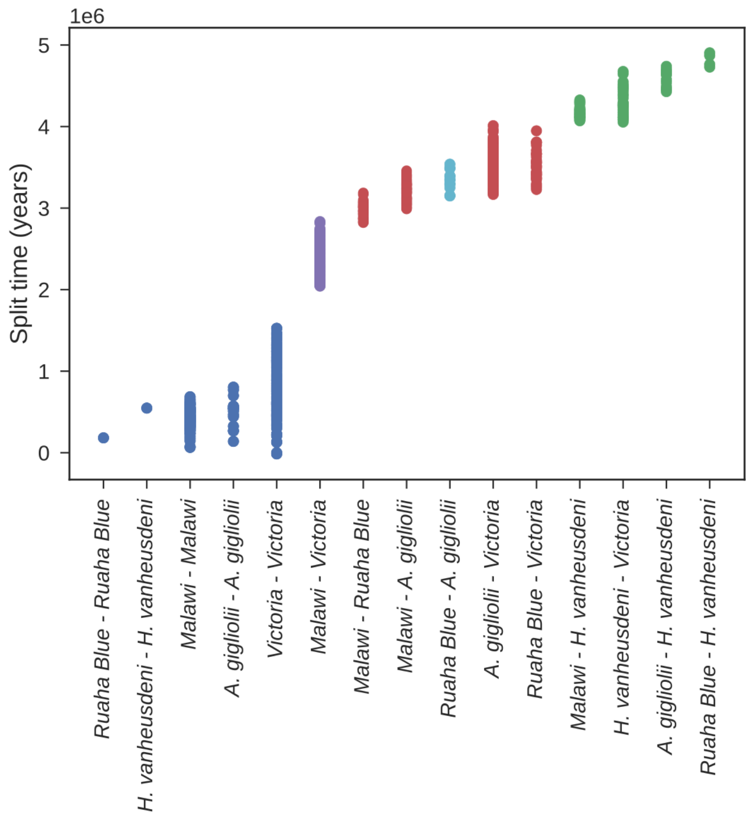

Pairwise sample split time estimates using equation (1), binned by clade, using a mutation rate of 3.5e-9 per bp per year (Malinsky et al. 2018) and a generation time of 3 years. Each dot corresponds to a point estimate obtained from a given pair of individual samples of the two clades on the x-axis. Blue dots correspond to within-group split time estimates. Other estimates are shown in the same colour if they correspond to the same split in fig. 1. Split time estimates assume the absence of gene flow between lineages. Note that mutation rate estimates are subject to uncertainty (Malinsky et al. 2018).

FIG. S7

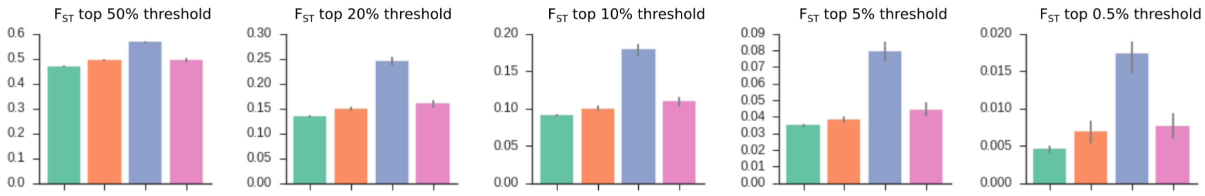

Proportion of F_{ST} outliers in the different SNP categories for different overall F_{ST} outliers thresholds on real data. Results for a top 1% threshold is shown in fig 3b.

FIG. S8

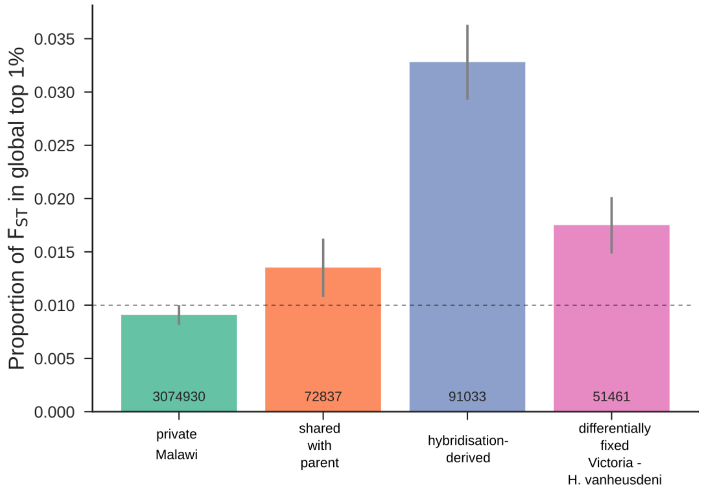

Distribution of F_{ST} outliers across variant categories described in Table 1. This plot is equivalent to fig. 3b, except that the *Victoria* category was subset to two samples, *Paralabidochromis flavus* and *Pundamilia nyererei*, so that it has the same sample size as *Ruaha Blue*.

FIG. S9

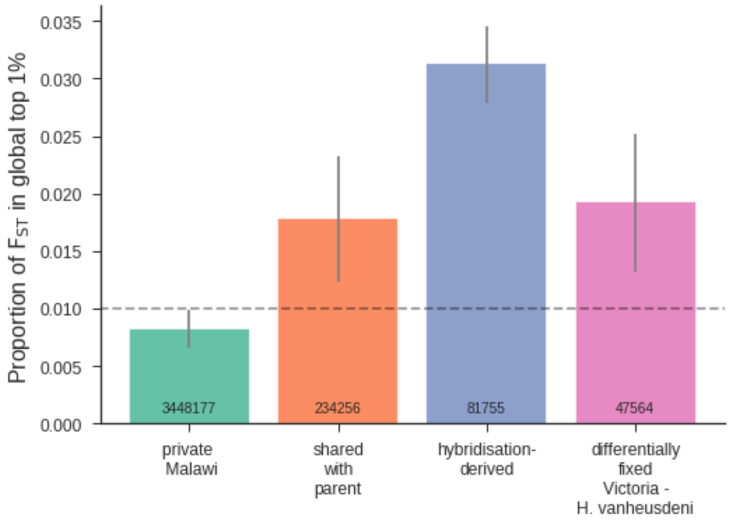

Distribution of F_{ST} outliers across variant categories described in Table 1. This plot is equivalent to fig. 3b, except done on a subset of samples that were realigned to a reference genome of *Astatotilapia calliptera* (GCA_900246225.3, https://www.ncbi.nlm.nih.gov/assembly/GCF_900246225.1/) (Materials and Methods) to confirm that results in fig. 3b are not an artifact of alignment to the relatively distant outgroup *O. niloticus*. Samples used in this analysis are tagged in supplementary table 1, column "A. calliptera alignment".

FIG. S10

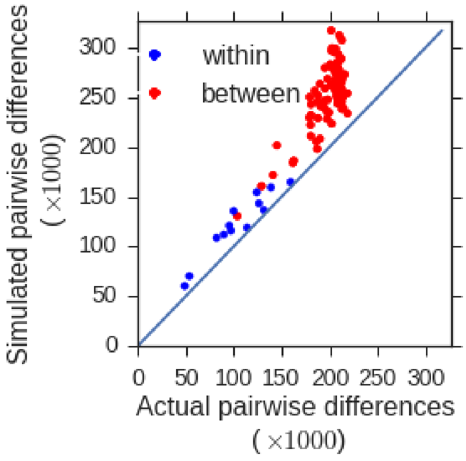

Comparison of pairwise differences between *Malawi* samples in the real data (x-axis) and simulations (y-axis).

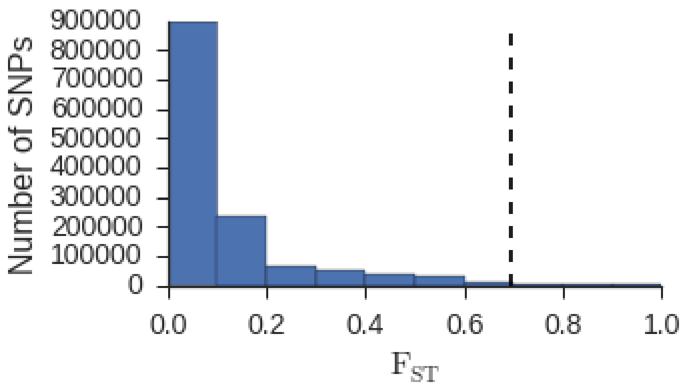

FIG. S11

Histogram of F_{ST} values for the pelagic-benthic split inferred from simulated data.

FIG. S12

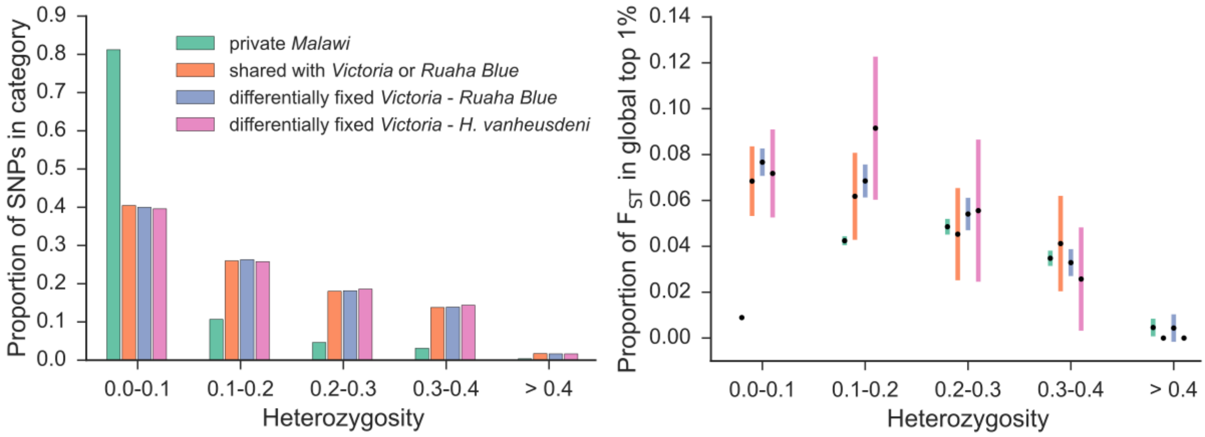

Distribution of F_{ST} outliers across variant categories on simulated data. For each variant category the proportion of SNPs in different heterozygosity bins is shown (left panel) . Heterozygosity is averaged across Malawi samples. Proportion of F_{ST} outliers in the variant categories described in table 1 stratified by heterozygosity (right panel). Error bars correspond to ± 3 block jackknifing standard deviations.

FIG. S13

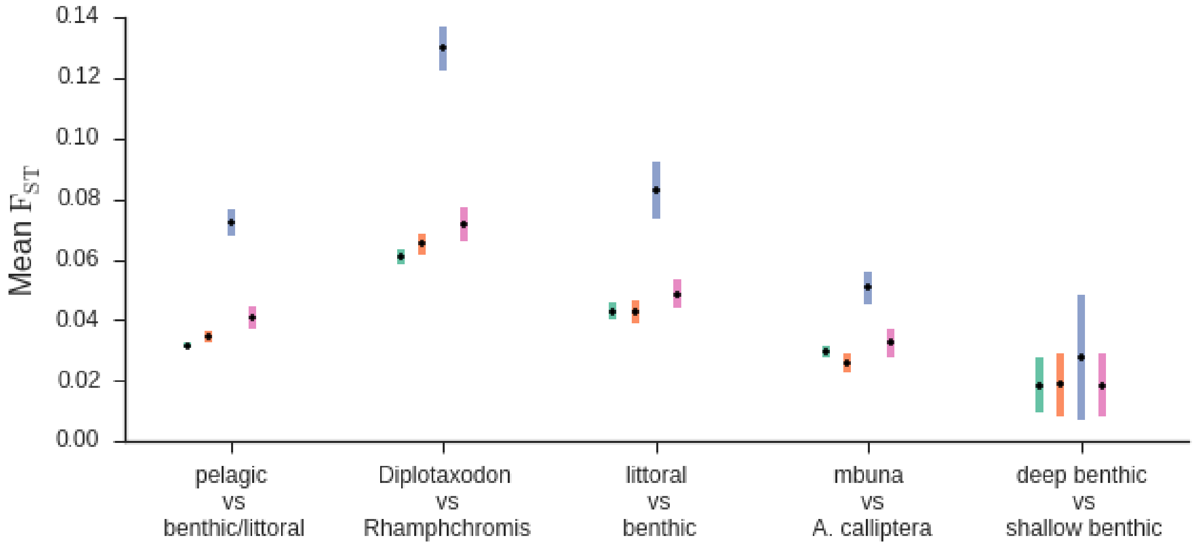

Mean F_{ST} in polymorphism categories for different splits between malawi eco-morphological groups. Color code as in fig. 3. Bars correspond to +/- three block jackknifing standard deviations.

FIG. S14

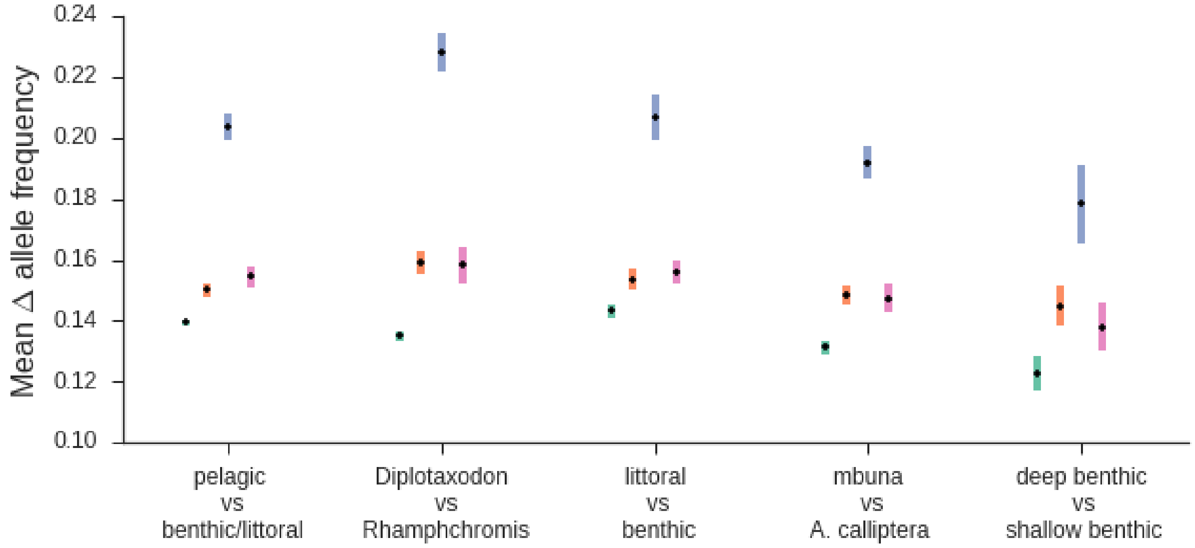

Mean absolute allele frequency difference in polymorphism categories for different splits between malawi eco-morphological groups. Color code as in fig. 3. Bars correspond to +/- three block jackknifing standard deviations.

FIG. S15

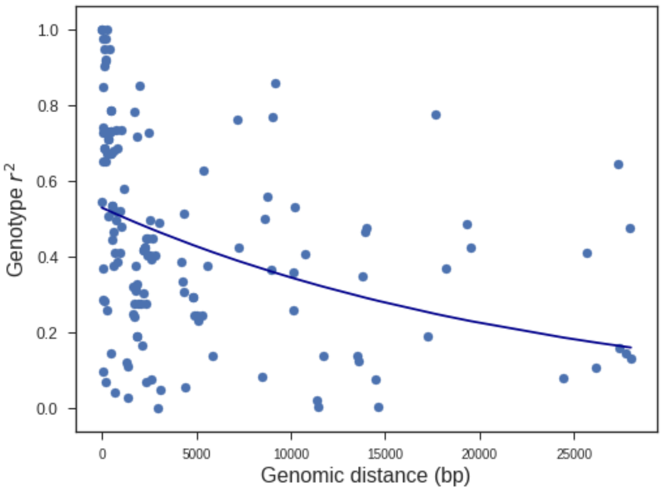

Genotype correlation of pairs hybridisation-derived variants in genes of the vision GO category as a function of genomic distance. Genotype correlation was calculated using the --geno-r2 option in vcftools (Danecek et al. 2011).

FIG. S16

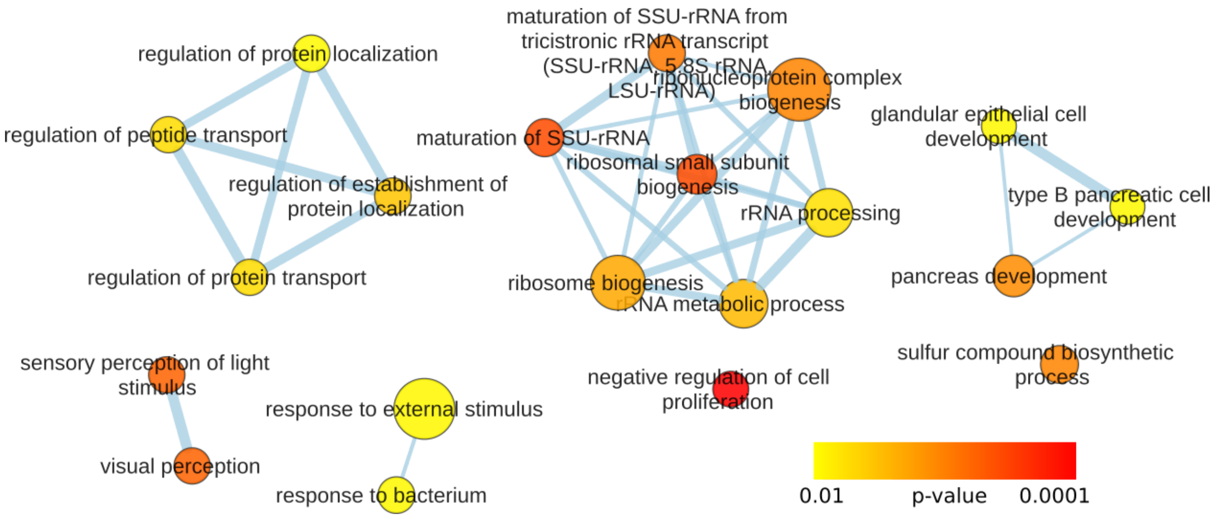

Enrichment of hybridisation-derived variants in biological process gene ontologies (GOs). Genes in the *Oreochromis niloticus* gene annotation were linked to zebrafish GO categories and enrichment of *Malawi* variants differentially fixed between *Victoria* and *Ruaha Blue* (category 3 in table 1) relative to

variants that are polymorphic in both Malawi and a parental lineage (category 2 in table 1) in exonic regions was tested (see Materials and Methods). Categories with enrichment p-values <0.01 are shown. Size of the circles is proportional to the number of genes present in the annotation in each category (from 10 to 220). This figure is equivalent to fig. 5 except for the normalisation with category 2 instead of category 1 variants.

FIG. S17

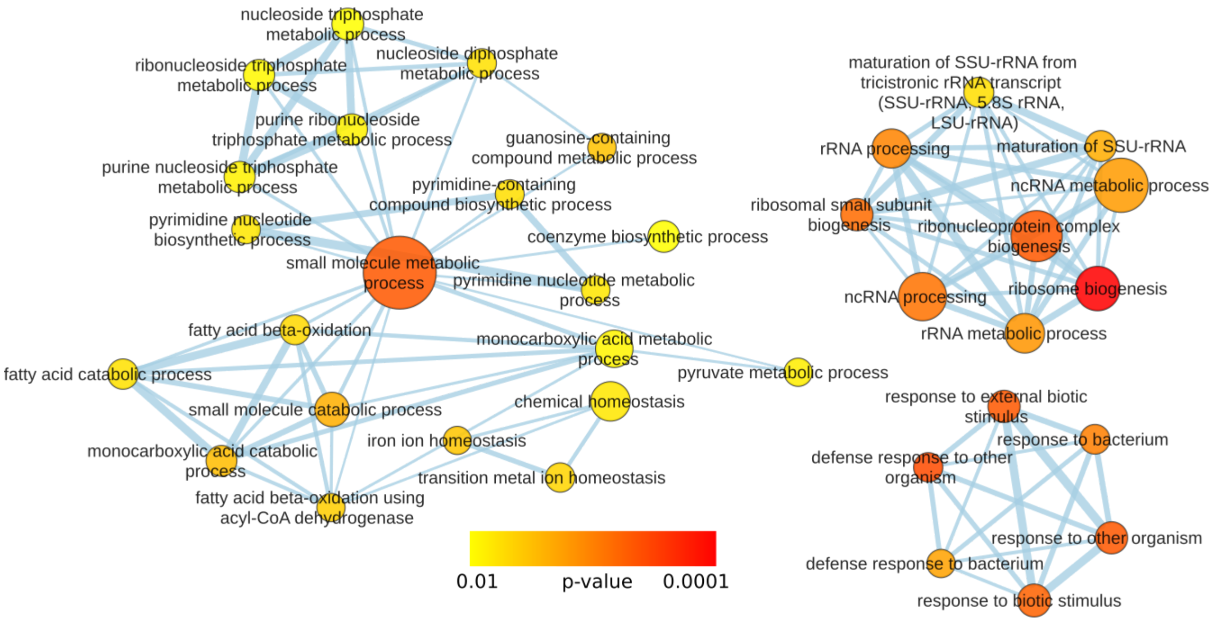

Enrichment of hybridisation-derived variants in biological process gene ontologies (GOs). Genes in the *Oreochromis niloticus* gene annotation were linked to zebrafish GO categories and enrichment of *Malawi* variants differentially fixed between *Victoria* and *Ruaha Blue* (category 3 in table 1) relative to accessible genome length in exonic regions was tested (see Materials and Methods). Categories with enrichment p-values <0.01 are shown. Size of the circles is proportional to the number of genes present in the annotation in each category (from 10 to 220). This figure is equivalent to fig. 5 except for the normalisation by accessible genome length instead of category 1 variants.

FIG. S18

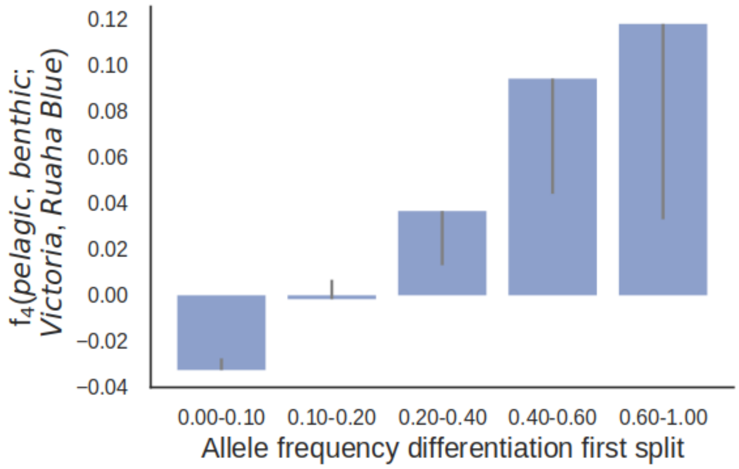

Allele sharing of lineages descending from *Malawi* first split with Victoria/*Ruaha Blue*. f_4 admixture ratio measuring the amount of *Victoria* ancestry in *benthic* relative to *pelagic* for hybridisation-derived variants. Error bars correspond to three jackknifing standard deviations and are only drawn towards the origin. Error bars that do not overlap the origin indicate to block-jackknifing significance p<0.01 (all bins except 0.10-0.20).

FIG. S19

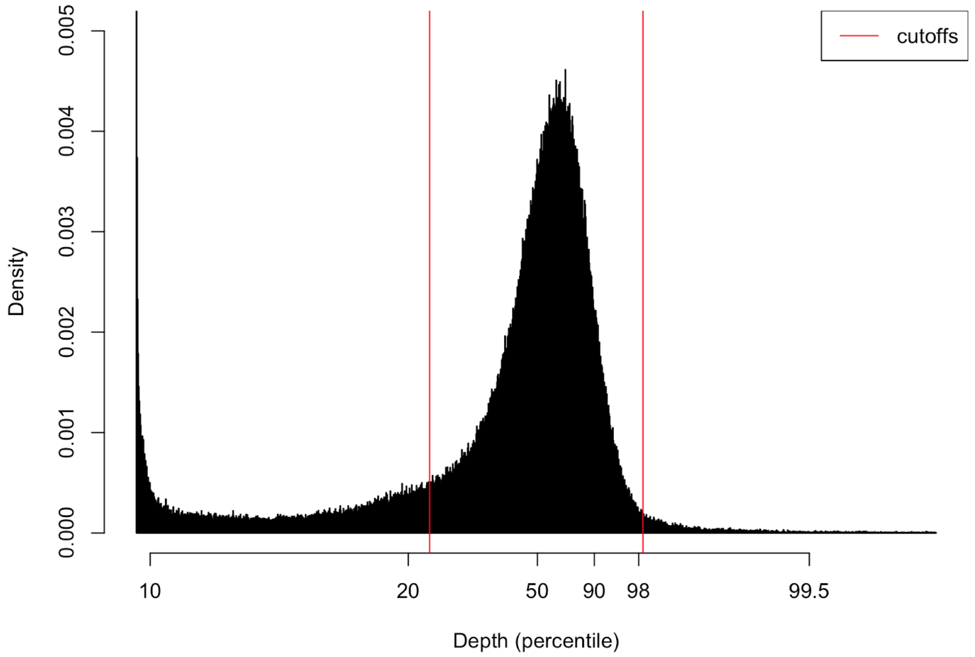

The cutoffs for filtering on overall depth (DP annotation in the VCF file) were selected by manually examining the distribution.

FIG. S20

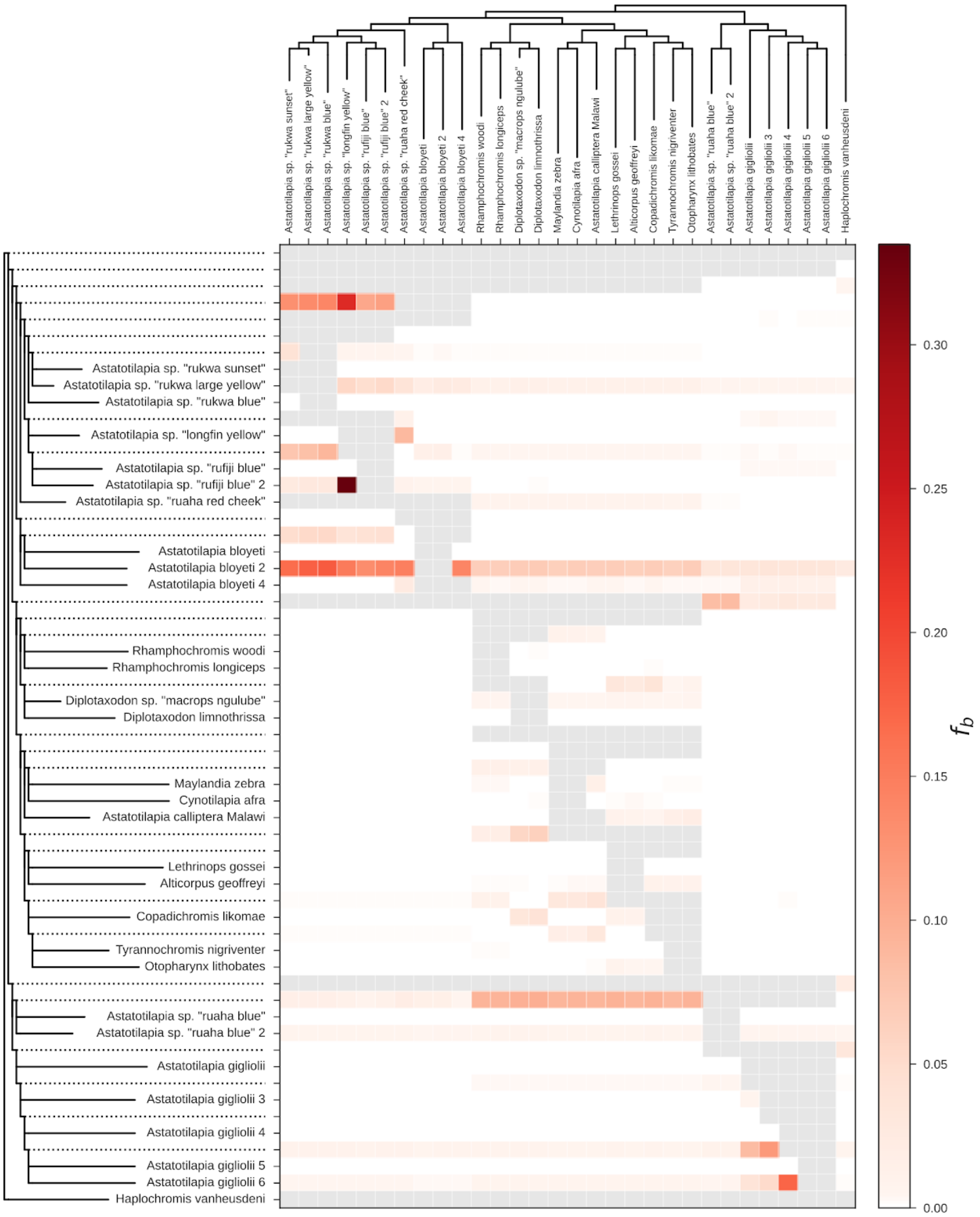

Excess allele sharing between samples. This figure is equivalent to supplementary fig. S3, but for a subset of the samples that was realigned to an A. calliptera reference genome (Materials and Methods). Results are consistent with supplementary fig. S3.

Supplementary Tables

Supplementary tables 1 and 2 are available as separate documents.

Supplementary table 1: Sample information, including NCBI accession numbers.

Supplementary table 2: All f4 admixture ratio tests (Patterson et al. 2012) that are consistent with the tree in fig. 1b for the outgroup (P4) being fixed as *Neolamprologus brichardi*.

References

Brawand D, Wagner CE, Li YI, Malinsky M, Keller I, Fan S, Simakov O, Ng AY, Lim ZW, Bezault E, et al. 2014. The genomic substrate for adaptive radiation in African cichlid fish. Nature. 513(7518):375–381.

Danecek P, Auton A, Abecasis G, Albers CA, Banks E, DePristo MA, Handsaker RE, Lunter G, Marth GT, Sherry ST, et al. 2011. The variant call format and VCFtools. Bioinformatics. 27(15):2156–2158.

Kelleher J, Etheridge AM, McVean G. 2016. Efficient Coalescent Simulation and Genealogical Analysis for Large Sample Sizes. PLoS Comput Biol. 12(5):e1004842.

Malinsky M, Svardal H, Tyers AM, Miska EA, Genner MJ, Turner GF, Durbin R. 2018. Whole-genome sequences of Malawi cichlids reveal multiple radiations interconnected by gene flow. Nat Ecol Evol. doi:10.1038/s41559-018-0717-x. http://dx.doi.org/10.1038/s41559-018-0717-x.

Patterson N, Moorjani P, Luo Y, Mallick S, Rohland N, Zhan Y, Genschoreck T, Webster T, Reich D. 2012. Ancient Admixture in Human History. Genetics. 192(3):1065–1093.
